# Wafer Bonding of GaAs and SiC via Thin Au Film at Room Temperature

**DOI:** 10.3390/mi16040439

**Published:** 2025-04-07

**Authors:** Kai Takeuchi, Eiji Higurashi

**Affiliations:** Graduate School of Engineering, Tohoku University, Sendai 980-8579, Japan; kai.takeuchi@tohoku.ac.jp

**Keywords:** thermal management, wafer bonding, semiconductor laser

## Abstract

Effective thermal management is a critical challenge in achieving high-power output for semiconductor laser devices. A key factor in laser device packaging is the bonding between the laser device on a GaAs substrate and a heat spreader, typically composed of high thermal conductivity materials such as SiC. Conventional soldering methods introduce thick bonding layers with relatively low thermal conductivity, resulting in high thermal resistance at the interface. In this study, we demonstrate the room temperature bonding of GaAs and SiC via a 30 nm thick Au layer, eliminating the need for a thermal reaction bonding layer or vacuum process. Using surface-activated bonding (SAB), GaAs and SiC were successfully bonded, with a strength comparable to bulk fracture. A uniform and ultrathin Au bonding interface significantly reduces thermal resistance compared to conventional soldering methods. These results highlight the potential of SAB with thin Au films as a promising approach for improving thermal management in high-power semiconductor laser devices.

## 1. Introduction

Due to their ability to deliver high optical power while maintaining excellent beam quality, semiconductor disk lasers (SDLs) have emerged as one of the most promising light sources. Among these, III-V semiconductor materials are widely utilized for high-power laser devices due to their superior optical and electronic properties. In particular, GaAs-based vertical external-cavity surface-emitting lasers (VECSELs) have garnered significant attention as high-power light sources for various applications, including optical communications [1,2], bioimaging [3,4,5], and sensing [6,7]. A key advantage of VECSELs is their tunable emission wavelength, typically spanning 850–1200 nm, enabling flexible adaptation to diverse technological applications.

To achieve both high optical power and precise beam control in a laser device, effective thermal management is crucial. Operating high-power lasers generates significant heat, leading to an increase in the active layer temperature. Due to thermal rollover effects, this temperature rise induces a wavelength shift and reduces output power [8,9]. Consequently, efficient heat dissipation is essential for ensuring stable operation and maintaining performance reliability.

Among the various thermal management strategies, integrating high-power laser structures with heat sinks is widely adopted, employing different materials and bonding configurations [10,11,12]. In the case of VECSELs, the distributed Bragg reflector (DBR) layers, which are typically integrated with the gain region, play a critical role in both optical feedback and thermal conduction. Since heat generated in the gain region must be dissipated through the DBR layers to the heat sink, conventional approaches rely on heat sink–laser chip bonding via soldering [13,14,15,16]. However, optimizing the thermal conductivity of this interface remains a key challenge in developing next-generation high-power VECSELs.

From a materials perspective, high thermal conductivity is a key requirement for heat sink and spreader materials in high-power semiconductor lasers. As a result, materials such as Cu with a thermal conductivity of 401 W/m·K [17], Al at 237 W/m·K [17], diamond at 2400 W/m·K [18], and SiC at 370 W/m·K [19] are commonly employed. Among these, SiC is particularly promising due to its high thermal conductivity, close thermal expansion match with GaAs, excellent thermal stability, and superior mechanical strength. Consequently, SiC-based heat sinks have been widely adopted in various high-power semiconductor laser applications [20,21].

However, the system’s thermal performance is strongly influenced by the bonding interface between the heat sink and the laser device. While soldering is a widely used bonding technique, the intrinsic thermal resistance of the solder layer limits heat dissipation efficiency. Furthermore, the typical solder layer thickness of 30–50 μm introduces additional thermal resistance, impeding effective heat transfer.

To optimize thermal management, the bonding layer should be as thin as possible while maintaining high thermal conductivity. In the case of AuSn soldering, a 3 μm solder thickness between the laser chip and heat sink has been reported to enhance thermal performance [22]. However, it has also been observed that reducing the solder thickness below 20 μm can lead to void formation at the bonding interface, potentially degrading thermal and mechanical reliability [23]. Consequently, achieving a submicron-thick solder layer for bonding between the laser chip and heat sink remains a significant challenge.

Recently, direct GaAs and SiC bonding using the surface-activated bonding (SAB) technique has been demonstrated [24]. With this method, substrate surfaces are activated using an Ar fast atomic beam (FAB) and subsequently bonded in a vacuum environment at room temperature [25,26,27]. As SAB enables direct bonding without forming a thermal reaction layer at the interface, residual thermal stress and damage to the devices can be reduced compared to conventional bonding methods with heating. However, the vacuum processing requirement limits its applicability in practical laser device packaging.

In the meantime, bonding via gold (Au) presents an attractive alternative for heat sink bonding due to its high thermal conductivity, excellent bondability, and compatibility with mild processing conditions, such as low bonding temperatures and pressures [28,29]. Notably, direct Au-Au bonding can be achieved at room temperature in ambient air using the SAB method [30,31,32,33]. In this process, Au surfaces are activated via RF plasma treatment, eliminating contaminants and creating highly reactive surfaces. The activated Au surfaces subsequently form metallic bonds upon contact, even in air. Since this technique does not involve thermal reactions at the interface, the resulting bonding layer can be as thin as ~10 nm, significantly reducing thermal resistance.

In this study, we explore the room temperature and in-air bonding of SiC and GaAs via Au SAB for the packaging of high-power VECSEL devices.

## 2. Methods

**Figure 1:**
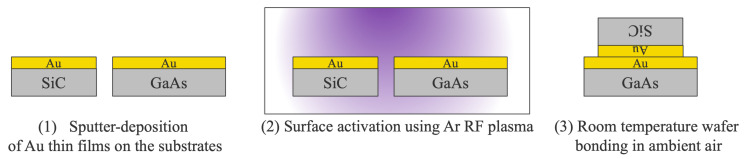
Bonding process of SAB via thin Au films.

The bonding process is illustrated in Figure 1. For the bonding experiments, we prepared 2-inch, 330 μm thick 4H-SiC and 3-inch, 450 μm thick GaAs wafers. Prior to bonding, the wafers were cleaned using a a two-fluid cleaning process to remove surface contaminants. Following the cleaning step, a 5 nm Ti adhesion layer and a 15 nm Au bonding layer were deposited on both the SiC and GaAs wafer surfaces via sputtering. After deposition, the Au surfaces were activated using RF plasma treatment at a power of 200 W for 30 s. Subsequently, the activated wafers were brought into contact at room temperature in ambient air to achieve direct bonding without bonding load. The bonding quality was evaluated based on surface roughness, bonding strength, and a cross-sectional analysis of the bonding interface.

## 3. Results and Discussion

**Figure 2:**
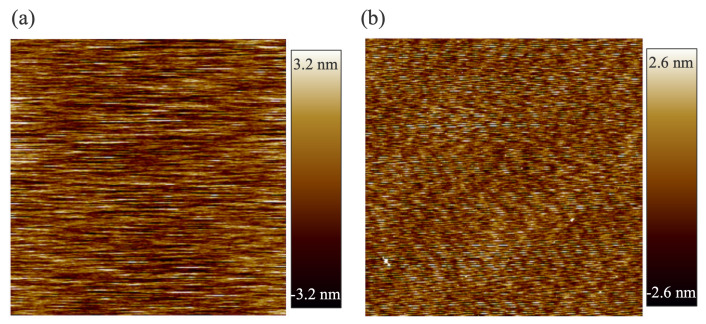
AFM images of the surfaces of (**a**) SiC and (**b**) GaAs wafers after Au deposition.

Atomic force microscopy (AFM) images of the SiC and GaAs surfaces after Au deposition are shown in Figure 2. The root mean square (RMS) surface roughness measured over a 10 × 10 μm^2^ area was 0.469 nm with a standard deviation of 0.073 (*n* = 4) for SiC and 0.460 nm a standard deviation of 0.059 (*n* = 4) for GaAs. Since SAB at room temperature requires an extremely smooth surface with a roughness of less than 1 nm, both SiC and GaAs wafers exhibit sufficiently smooth surfaces suitable for bonding.

**Figure 3:**
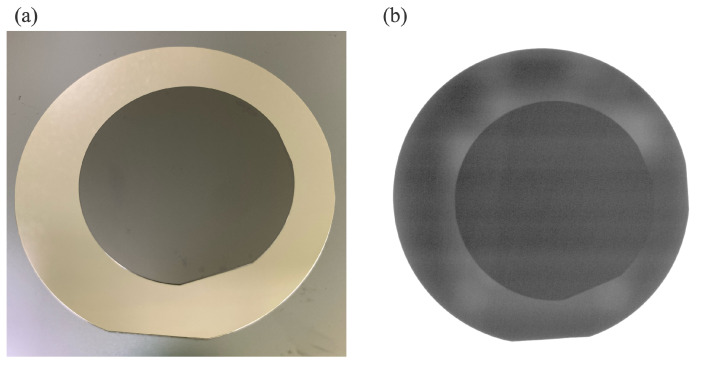
(**a**) Optical photograph of the bonded three-inch GaAs wafer and two-inch SiC wafer. (**b**) IR image of the bonded wafers.

The bonded SiC and GaAs wafers are shown in Figure 3. Figure 3a presents an optical photograph of the bonded wafers, while Figure 3b shows an infrared (IR) image of the bonded region. The IR image confirms that the entire 2-inch SiC wafer is completely bonded to the 3-inch GaAs wafer without observable defects. As the interfacial voids can be detected with interference fringes in previous study [30], the results indicates a successful bonding process.

**Figure 4:**
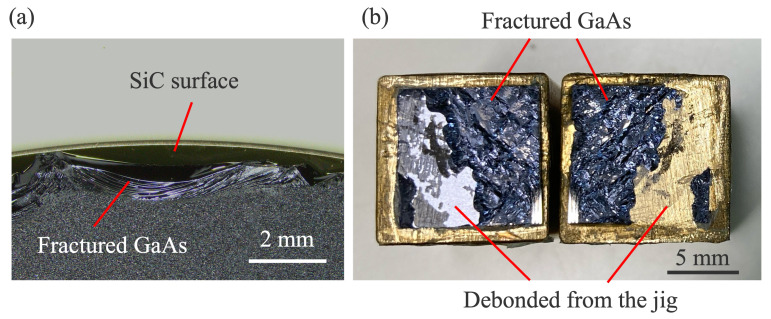
Micrographs of (**a**) the fractured GaAs wafer from the blade insertion test and (**b**) the fractured GaAs substrate from the tensile test.

Bonding strength was evaluated through blade insertion and tensile tests. As shown in Figure 4a, the GaAs wafer fractured during the blade insertion test, indicating that the bonding strength exceeds the surface free energy of GaAs (0.86 J/m^2^ [34]). For the tensile test, 1 × 1 cm^2^ chips were diced from the bonded wafers. The tensile strength was measured to be 14.9 MPa, and fracture occurred in the GaAs substrate, as shown in Figure 4b. This confirms that the bonding strength between GaAs and SiC exceeds 14.9 MPa. Both mechanical tests demonstrate that the bonded interface is mechanically robust.

**Figure 5:**
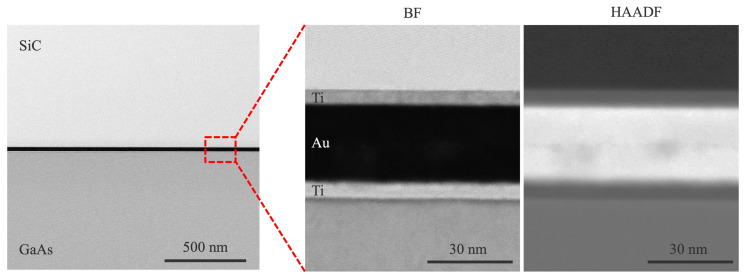
Cross-sectional TEM image of the bonding interface between SiC and GaAs via a thin Au layer.

Figure 5 presents cross-sectional transmission electron microscopy (TEM) images of the GaAs/SiC bonding interface. The low-magnification image confirms the uniformity of the bonded interface, while the high-magnification image reveals that GaAs and SiC are bonded via a 5 nm Ti adhesion layer and a 27 nm Au bonding layer. These results indicate that successful bonding was achieved at room temperature.

**Figure 6:**
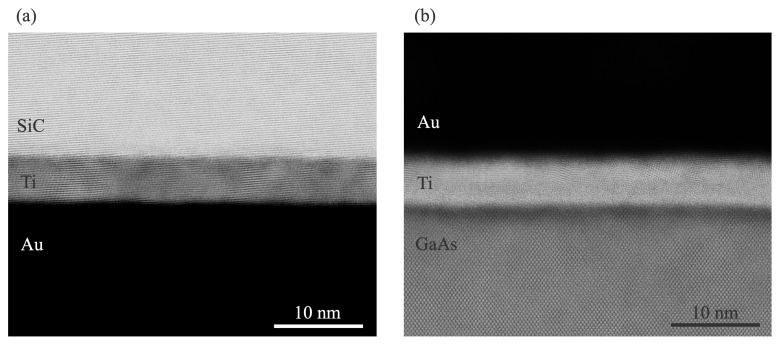
Magnified TEM images of the interfaces (**a**) between the SiC substrate and Au layer and (**b**) the Au layer and GaAs substrate.

Further magnified TEM images of the Au/SiC and Au/GaAs interfaces are shown in Figure 6. The images reveal that the crystalline structures of SiC and GaAs remain intact at the outermost wafer surfaces, indicating that room-temperature bonding was achieved without inducing structural damage. While previous studies have reported surface damage due to the surface activation process in direct SiC/GaAs bonding [24], the use of Au-mediated SAB effectively suppresses such damage, preserving bonded bonded materials.

**Figure 7:**
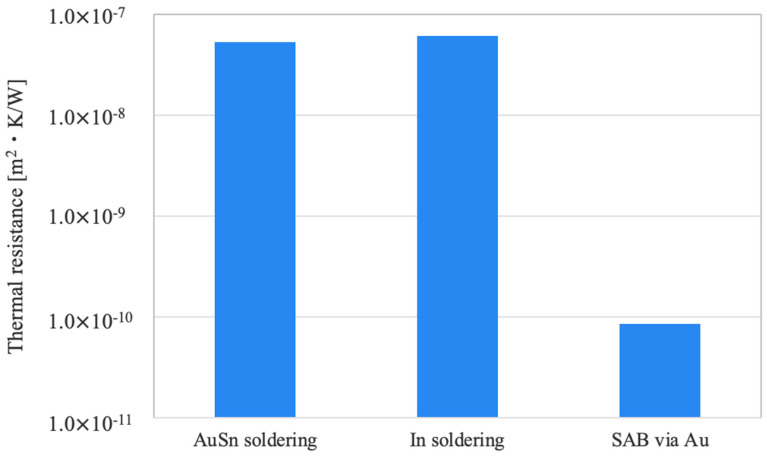
Estimated thermal resistance of the conventional AuSn soldering interface and the SAB bonding interface via thin Au films.

Based on the TEM results, we estimated the thermal resistance of the bonding layer formed by SAB using thin Au films. Figure 7 compares the calculated thermal resistance of a conventional AuSn solder bonding interface with that of the SAB utilizing thin Au films. Calculations were performed using thermal conductivities of 57 W/m·K for AuSn [35], 82 W/m·K for In [17], and 317 W/m·K for Au [17], with bonding layer thicknesses of 3 μm [22], 5 μm [14,15] and 27 nm, respectively, as can be determined in Figure 5. The estimated thermal resistances for the conventional AuSn and In soldering interface are 5.26 × 10^−8^ m^2^·K/W and 6.10 × 10^−8^ m^2^·K/W, whereas the SAB bonding interface via thin Au films achieves an impressively low thermal resistance of 8.60 × 10^−11^ m^2^·K/W.

This reduction of three orders of magnitude is attributed to the superior thermal conductivity of Au and the ultrathin bonding layer. These results demonstrate that SAB using thin Au films not only provides high bond strength but also significantly enhances thermal management efficiency. In addition, it is known that solder bonding has creep failure due to the thermal cycling-induced stress [36]. As the proposed bonding via SAB with Au is solid-state bonding, it is expected that the SAB bonding interface has higher long-term reliability compared to liquid-phase bonding such as soldering. Therefore, this technique is highly promising for the integration of GaAs and SiC in high-power semiconductor laser devices.

## 4. Conclusions

In this study, GaAs and SiC wafers were successfully bonded at room temperature in ambient air using SAB with thin Au films. The SAB process resulted in a high bond strength and excellent bonding quality. Cross-sectional TEM analysis confirmed the formation of a uniform Au bonding interface without inducing damage to the wafer surfaces. Furthermore, the estimated thermal resistance of the SAB bonding interface was significantly lower than that of conventional solder bonding, highlighting its superior thermal management capability. These findings demonstrate the potential of SAB with thin Au films in advanced packaging for high-power semiconductor laser devices.

## Data Availability

The datasets used and/or analyzed during the current study can be made available from the corresponding author upon reasonable request.
